# Influence of time between surgery and postoperative radiation therapy and total treatment time in locoregional control of patients with head and neck cancer: a single center experience

**DOI:** 10.6061/clinics/2020/e1615

**Published:** 2020-07-15

**Authors:** Rejane Franco, Leandro Luongo de Matos, Marco Aurélio Vamondes Kulcsar, Gilberto de Castro-Júnior, Gustavo Nader Marta

**Affiliations:** I Programa de Pos-Graduacao, Departamento de Radiologia e Oncologia, Instituto do Cancer do Estado de Sao Paulo (ICESP), Hospital das Clinicas HCFMUSP, Faculdade de Medicina, Universidade de Sao Paulo, Sao Paulo, SP, BR; II Hospital de Clinicas, Universidade Federal do Parana, Curitiba, PR, BR.; III Departamento de Cirurgia de Cabeca e Pescoco, Instituto do Cancer do Estado de Sao Paulo (ICESP), Hospital das Clinicas HCFMUSP, Faculdade de Medicina, Universidade de Sao Paulo, Sao Paulo, SP, BR; IV Unidade Clinica de Oncologia, Departamento de Radiologia e Oncologia, Instituto do Cancer do Estado de Sao Paulo (ICESP), Hospital das Clinicas HCFMUSP, Faculdade de Medicina, Universidade de Sao Paulo, Sao Paulo, SP, BR; V Departamento de Oncologia Clinica, Hospital Sirio-Libanes, Sao Paulo, SP, BR; VI Departamento de Radiologia e Oncologia, Servico de Radioterapia, Instituto do Cancer do Estado de Sao Paulo (ICESP), Hospital das Clinicas HCFMUSP, Faculdade de Medicina, Universidade de Sao Paulo, Sao Paulo, SP, BR; VII Departamento de Radioterapia, Hospital Sirio-Libanes, Sao Paulo, SP, BR

**Keywords:** Head and Neck Neoplasms/Therapy, Head and Neck Neoplasms/Surgery, Prognosis, Adjuvant Chemoradiotherapy

## Abstract

**OBJECTIVE:**

This study aimed to evaluate the effect of the delay to initiate postoperative radiation therapy (RT) on locoregional control to head and neck squamous cell carcinoma patients.

**METHODS:**

Retrospective cohort study that included patients submitted to surgery followed by adjuvant RT (with/without chemotherapy). The time interval between surgery and RT was dichotomized by the receiver operating characteristics curve method at 92 days. Other possible sources of heterogeneity with potential impact on locoregional control were explored by regressive analysis.

**RESULTS:**

A total of 168 patients were evaluated. The median time for locoregional recurrence (LRR) was 29.7 months. The relapse-free survival rates were 66.4% and 75.4% for patients who initiated RT more than and within 92 postoperative days (*p*=0.377), respectively. Doses lower than 60Gy were associated with worse rates of locoregional control (HR=6.523; 95%CI:2.266-18.777, *p*=0.001). Patients whose total treatment time (TTT) was longer than 150 days had LRR rate of 41.8%; no patient with TTT inferior to 150 days had relapses (*p*=0.001).

**CONCLUSIONS:**

The interval between surgery and RT did not show influence on locoregional control rates. However, doses <60Gy and the total treatment time >150 days were associated with lower locoregional control rates.

## INTRODUCTION

Two-thirds of all head and neck squamous cell carcinoma (HNSCC) patients are diagnosed at advanced clinical stages, which is related to a dismal prognosis (1,2). The management of these patients with locally advanced disease must include a multimodal strategy including surgery, radiation therapy (RT), and systemic therapies (3). Although surgical resection with curative intent followed by postoperative RT has been one of the recommended treatments for most patients with locally advanced tumors, it is associated with unsatisfactory outcomes, such as 30% of locoregional failures, 25% of distant metastases, and 5-year survival rates of 50% (4-6).

Beyond clinical and tumor-related characteristics, treatment-related variables may also have a prognostic impact on clinical outcomes, such as dosing, duration, and timing to initiate postoperative RT (7). The delay to initiate postoperative RT mainly seems to affect patients with accelerated cell proliferation tumors such as HNSCC (8). This possible deleterious effect is based on the rationale that the doubling time and the tumor growth rate are directly related to the locoregional control of the neoplasia (9). It is not unusual, however, some delay before starting adjuvant therapy: 1 out of 4 patients has prolonged intervals between surgery and RT due to difficulties in accessing treatment centers, prolonged hospitalization, surgical recovery or postoperative complications (10). The recommendation regarding treatment time for head and neck cancer patients according to the globally used National Comprehensive Cancer Network guideline consists of an interval between surgical resection and the postoperative RT preferably shorter than 6 weeks (11). However, among the variables that compose the adjuvant timeline treatment (RT length, interval between surgery and RT onset, or total treatment time from surgery to the last day of RT), a lack of consensus does exist about the interval time between surgery and postoperative RT (12,13) with unconformity between several studies that analyzed this interval, some of them reporting an important association with local control and survival (8,9,13-17) and others not (7,12,18-20).

The present study aimed to evaluate the effect of the delay in initiating postoperative RT on locoregional outcomes of patients with HNSCC.

## MATERIALS AND METHODS

This is a retrospective cohort study with data collected from electronic medical records to assess demographic and treatment characteristics of the patients. Patients included were those diagnosed with HNSCC without distant metastasis who underwent surgical resection with curative intent followed by postoperative RT, with or without adjuvant chemotherapy, from January 2009 to January 2015 at a single institution. Only patients without gross recurrent disease before postoperative treatment were included.

The exclusion criteria included lack of relevant data to the analysis and/or gross residual disease after surgical treatment.

This study was approved by the local institutional ethical review committee.

### Adjuvant Treatment Protocol

#### Radiation Therapy

Patients referred to postoperative RT were those who presented pathological factors classifying them as high risk for local recurrence (locally advanced tumors pathologically staged as III/IV, positive involvement of regional lymph nodes, and positive surgical margins but not restricted to perineural invasion or angiolymphatic invasion).

RT was performed using conformal technique with a linear megavoltage accelerator. We applied the recommendations for treatment dose and volumes and constraints for organs at risk as previously reported (4). The prescription doses varied between 60-66 Gy at high-risk areas/surgical bed and 50 Gy at elective undissected nodal volume deemed at risk of harboring microscopic disease, 1 fraction per day, 5 times per week. Low RT doses (<60Gy) were administered to patients whose clinical condition required early treatment interruption, whether due to performance deterioration or local progression. No alternative fractionations such as accelerated fractionation or hypofractionation were employed.

#### Chemotherapy

Patients with positive surgical margins and/or nodal extracapsular extension were candidates for adjuvant chemotherapy, consisting of cisplatin (100 mg/m^2^ intravenously every 21 days) concurrently to RT

### Statistical Analysis

The endpoint analyzed was the locoregional control rate, considered as the fraction of patients presenting freedom from local or regional progression at the moment of data analysis.

The values obtained of each quantitative variable of parametric distribution were organized and described by median and standard deviation. Absolute and relative frequencies were used for the qualitative variables. The receiver operating characteristics (ROC) curve method was used to determine cutoff values for risk stratification for quantitative variables. The Kaplan-Meier method was used for the univariate survival analysis and the log-rank test was used for the comparison between curves. The variables with *p*<0.10 at univariate analysis were submitted to the Cox regression model with a hazard ratio (HR) 95% confidence interval (95% CI) at multivariate analysis. Due to the retrospective design of the study, which is subjected to biases inherent to the method, possible sources of heterogeneity were explored through regression analysis, taking into account the potential effect of other factors on locoregional control. Statistical significance was less than 5% (*p*≤0.05) in all analyses, using the statistical program SPSS version 17.0 (SPSS Inc., Illinois, USA).

## RESULTS

### Clinical and Pathological Features

During the study, data were collected from 193 consecutive patients, with 168 patients being included in the analysis. Fifteen patients were excluded because of missing records such as date of surgery or date of RT onset; 10 patients were excluded because they presented gross residual disease after surgery. The majority were male (n=132, 78.6%), with oral cavity cancer (n=95; 56.5%), and a median age of 62 years (range 41-92 years). About 93% of patients had locally advanced disease stage III or IV, according to the seventh edition of the American Joint Commission on Cancer staging system.

Surgical margins were negative in 86.9% and lymph nodes were pathologically involved in 66.7%. Among the patients with positive lymph nodes (n=112), 32.7% had extracapsular spread. In addition, 32.7% of the patients had lymphovascular invasion and 70.8% had perineural invasion (Table 1).

### Therapeutic Characteristics

Among 168 patients analyzed, 80 (47.6%) received adjuvant chemotherapy concurrently with RT. Most of them (95.2%) received doses of 60-66 Gy at surgical bed/high-risk areas and 50 Gy at elective lymph node areas. The median waiting time for consultation with a radiation oncologist since surgery was 63.3 days (range 13-182 days). The median time from RT consultation to the beginning of postoperative treatment was 52.7 days (range 1-202 days), and the median time interval from surgery to the RT initiation was 116.1 days (range 40-250 days). The median course of RT lasted 51 days (range 14-103 days), with the majority of patients presenting interruptions during RT (n=115, 68.5%), with no clinical reason identified, and an average of 4 days of absence (range 0-42 days). The majority of patients (n=124; 73.9%) did not present postoperative complications and had a median total treatment time (TTT) of 167.2 days (range 88-322 days).

The interval to start RT from the surgical date was dichotomized between patients who had RT started before and after 92 days postoperatively. This cutoff was determined based on the value assigned to the sensitivity of 21.15 (95% CI 11.1-34.7) and the specificity of 70.87 (95% CI: 62.1-78.6) by the ROC curve method for risk stratification.

### Clinical Outcomes

**Figure 1:**
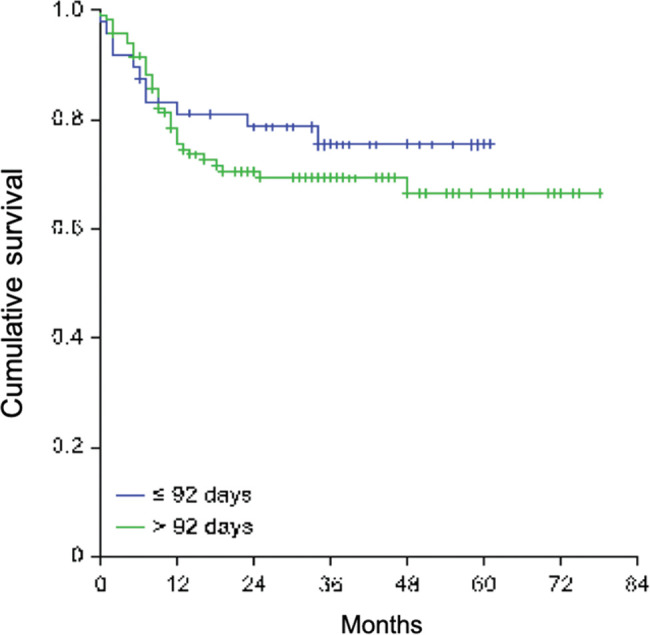
Kaplan-Meier curves of cumulative survival free from relapse according to time to start of RT - cutoff at 92 days.

With a median follow-up time of 33 months (5-78 months) since the date of surgery, the locoregional control rate for the entire cohort was 72.6%, with a median time for locoregional recurrence of 29.7 months (range 0-78 months). The univariate analysis detected the following factors related to locoregional recorrence: angiolymphatic invasion (*p*=0.055), perineural invasion (*p*<0.001), regional lymph nodes involvement (*p*=0.001), extracapsular nodal extension (*p*=0.014), tumor bed dose <60Gy (*p*=0.03), and KPS <70 before RT (*p*=0.001; Table 2). The relapse-free survival rate was 66.4% for patients who started RT with more than 92 post-operative days *versus* 75.4% for those who started RT within 92 days (*p*=0.377), as shown in Figure 1.

At multivariate analysis (Table 3), KPS <70 (HR=2.058, 95%CI:1.060-3.992, *p*=0.033), tumor bed dose <60Gy (HR=6.523; 95% CI: 2.266-18.777; *p*=0.001), positive lymph nodes (HR=3.339; 95%CI:1.350-8.255; *p*=0.009), and perineural invasion (HR=3.529; 95%CI:1.236-10.074; *p*=0.018) were independent variables related to worse locoregional control.

**Figure 2:**
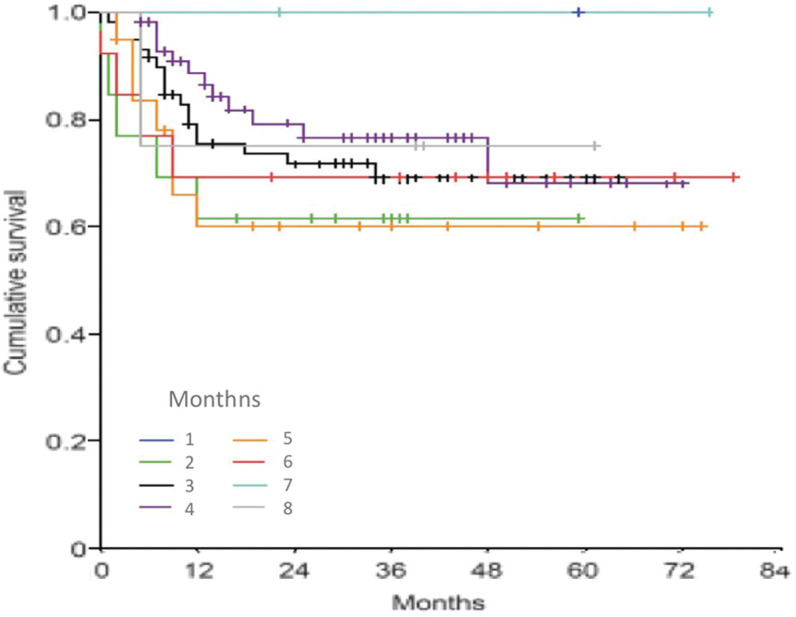
Kaplan-Meier curves of cumulative survival free from relapse in relation to time to start of RT - cutoff from 1 to 8 months.

At subanalysis performed in order to detect a possible time threshold related to better locoregional control, inferior or superior to the 92-day cutoff previously analyzed, monthly intervals between 1 and 8 months were stratified without any of them being related to worse rates of tumor control (Figure 2).

Taking into account the potential effect of other factors on locoregional outcomes, we explored by regressive analysis possible sources of heterogeneity. When evaluating the impact of surgical margins on locoregional control rates, we did not find influence at univariate nor multivariate analysis. Among patients presenting positive surgical margins, the interval to start adjuvant RT did not show significance on locoregional control rates, with 64.3% for those whose interval was >92 days and 62.5% with interval <92 days (*p*=0.95) (Appendix - Figure 1S).

The addition of adjuvant chemotherapy to RT in those patients with delay >92 days to initiate postoperative RT revealed a trend of better locoregional control rates: 76.8% for the combined treatment versus 65.6% in those patients receiving postoperative RT alone (*p*=0.056).

Primary site did not present a statistically significant impact on locoregional control rates, although laryngeal tumors presented the lowest local recurrence rate in relation to others sites at univariate analysis (Appendix - Figure 2S and Table 2). When we analyzed the relationship between the delay >92 days to start adjuvant treatment according to each individual tumoral site, we did not find an association between a prolonged interval and the locoregional recurrence rates at any particular anatomical subsite.

**Figure 3:**
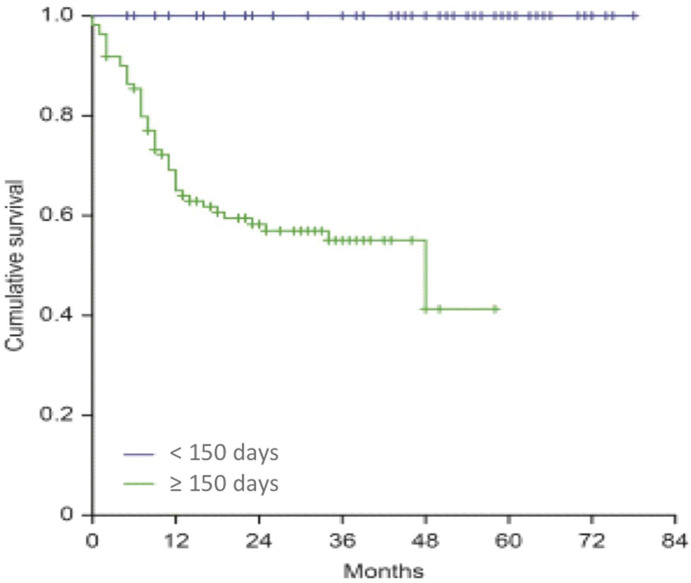
Kaplan-Meier curves of recurrence-free survival according to the total treatment time - cutoff at 150 days.

The variable that provided an interesting association in relation to the locoregional control was the TTT. We observed for 110 patients with a TTT greater than 150 days a locoregional recurrence rate of 41.8%, whereas no relapses occurred among patients with treatment duration inferior to 150 days (*p*=<0.001), as shown in Figure 3.

## DISCUSSION

Postoperative RT is usually administered 5 times per week over 5 to 7 weeks. The radiobiological principle inherent to this schedule is to allow, during the interval time between fractions, recovery to normal tissues from sublethal injuries caused by radiation, allowing the renovation of damaged healthy cells. Nevertheless, fractionation also enables repopulation of the surviving tumor cells (21), a phenomenon defined as repopulation (22). Thus, when prolonged intervals between fractions occur, accelerated repopulation can be pronounced and the effectiveness of RT can be compromised (23). In order to quantify the percentage of patients who were able to follow the National Comprehensive Cancer Network’s recommendation and started RT within 6 weeks postoperatively (11), Ho et al. (24) evaluated a database of 15 064 patients and Graboyes et al. (13) evaluated 41,291 patients with HNSCC submitted to surgical resection followed by postoperative RT. They found that more than 50% of patients did not succeed at initiating RT within 6 weeks postoperatively, almost 40% of them did not initiate treatment within 7 weeks and 30% of the patients had an interval greater than 8 weeks. The authors showed that the main factors related to the delay to initiate RT included severe clinical comorbidities, low socioeconomic status, postoperative complications, need for a new surgical approach, oncological treatment at academic hospitals, prolonged waiting time for pathological surgical report, fragmented treatment with surgery and RT at different hospitals, and use of RT with sophisticated intensity modulated technique. Similarly, most of the patients analyzed by the present study presented a delay to start adjuvant therapy, with 71.4% presenting a time interval greater than 92 days. Despite the predominant delay to initiate postoperative RT, both locoregional control rate (72%) and median time to relapse (29 months) were similar to those reported in the previous series of HNSCC patients treated with surgery and postoperative RT (25-27). The fact that the present study did not find association between locoregional control and the delay to start RT, even though most patients had a long interval to initiate adjuvant treatment, may be related to the influence of other prognostic factors on therapeutic outcomes. The Memorial Sloan Kettering Cancer Center group endorses this assumption by showing the influence of treatment RT dose when most of the locoregional recurrences occurred in patients who had received RT doses lower than 60 Gy despite starting postoperative RT within 6 weeks. The different locoregional control rates were also attributed to the suboptimal dose rather than to the treatment delay. According to the authors, a possible delay to initiate postoperative RT would not have an effect on outcomes when appropriate tumoricidal doses are administered (20). In our sample, 95.2% of the patients had received at least 60 Gy to the tumor bed and a median treatment dose of 62 Gy, with doses lower than 60 Gy being associated with worse rates of locoregional control either at uni or multivariate analysis (HR=6.523; 95%CI:2.266-18.777, *p*=0.001). However, we must recognize a possible bias, since patients who received doses lower than 60 Gy were those who presented deteriorated performance status and/or early local disease progression.

The Intergroup 0034 (28) and RTOG 0024 (29) studies evaluated the effect of adjuvant chemotherapy on patients with HNSCC, the first by administering adjuvant chemotherapy to patients who would initiate RT 4 months after surgical treatment and the second by providing early chemotherapy prior to adjuvant RT onset. They demonstrated that chemotherapy is able to decrease accelerated cell repopulation and could contribute to minimizing possible deleterious effects related to RT delay. These results are in consonance with the present study, which demonstrates a tendency for lower rates of locoregional recurrence for patients with postoperative interval greater than 92 days who received adjuvant chemotherapy compared to those with prolonged interval treated exclusively with postoperative RT.

The vast majority of studies were conducted analyzing different HNSCC subsites together, although evidence showing heterogeneous biological behavior according to tumor subsite exists. Among the studies that evaluated the influence of time interval between surgery and RT onset, few of them presented analysis according to a single anatomical tumor subsite (17). In the present study, a subgroup analysis related to the tumor site was performed and no differences were observed among the different sites, because most patients included had oral cavity cancer.

Although this study had focused on the influence of time interval to start postoperative RT, it is important to highlight the findings related to TTT, without any locoregional recurrence when TTT lasted up to 150 days. Published data corroborate with this finding by, similarly, presenting worse results matching the longer TTT duration. A collaborative study between UTMDACC, the H Lee Moffitt Cancer Center, and the Mayo Clinic (16) showed that prolonged interval between surgery and postoperative RT had a significant impact on locoregional and survival rates of patients who received RT with conventional fractionation, but it did not affect patients receiving RT with accelerated fractionation, suggesting that total combined treatment duration significantly affects local control and survival rates, recommending the TTT to have the shortest possible duration.

Sanguineti et al. (30) in a prospective multi-institutional study, randomized patients to receive 60 Gy over 6 weeks versus 64 Gy over 5 weeks and did not find any statistically significant difference in terms of locoregional control between the 2 groups (80% *vs* 78%; *p*=0.52). However, in a subgroup analysis restricted to patients who presented delay before starting postoperative RT (interval longer than 7 weeks at the study), they found a trend toward better rates of locoregional control with the shortest RT treatment (85% *vs* 71%; *p*=0.13). The authors concluded that RT in an accelerated fractionation should be considered for patients with delay before starting postoperative RT.

Parsons et al. (17) evaluated the influence of intervals that comprise the treatment of patients with oral cavity tumor submitted to surgical resection followed by postoperative RT. They did not find impact related to the duration of RT nor the interval between surgery to RT onset, but they detected better locoregional control rates when the total treatment duration did not exceed 100 days (14% *vs* 60%, *p*=0.04). Rosenthal et al. (18) retrospectively evaluated the importance of the variables that make up the TTT of patients with head and neck cancer submitted to surgery followed by postoperative RT. They did not detect statistical differences at the individual components of treatment, but they attributed better locoregional and overall survival rates for patients who completed the TTT within 100 days.

Although the TTT is composed by independent variables, we consider that it should be evaluated as components of a single unit and the treatment analyzed globally through its collective and not individual impact, with the attention concentrated in every stage, and not only at the interval between surgery and RT.

Our study diverges considerably from other studies previously published by presenting a markedly prolonged time to initiate RT. This was not on purpose, but related to resource restrictions intrinsic to our public health care system that precluded timely delivery of RT. These features, nevertheless, offered a single chance to study the potential of a significantly extended time to initiate RT on local control rates in HNSCC patients. Furthermore, the literature, based of phase 3 trials, does not present a clearly cutoff time to initiate RT that would indicate a critical detrimental outcome, which was heartening. Luckily, the logistics difficulties are being improved, and presently, we are working with a time to initiate RT of 6 weeks.

In conclusion, the interval between surgery and RT did not show influence on locoregional control rates for patients with head and neck cancer. However, doses <60Gy and the total treatment time >150 days were associated with lower locoregional control rates.[Fig f04][Fig f05]

## AUTHOR CONTRIBUTIONS

Franco R was responsible for the data curation, investigation, and writing original draft. Matos LL was responsible for the formal analysis, methodology and validation. Kulcsar MAV was responsible for the investigation, formal analysis and validation. Castro-Júnior G was responsible for the investigation, supervision, and manuscript writing, editing and review. Marta GN was responsible for the conceptualization, project administration and supervision.

## Figures and Tables

**Figure 4 f04:**
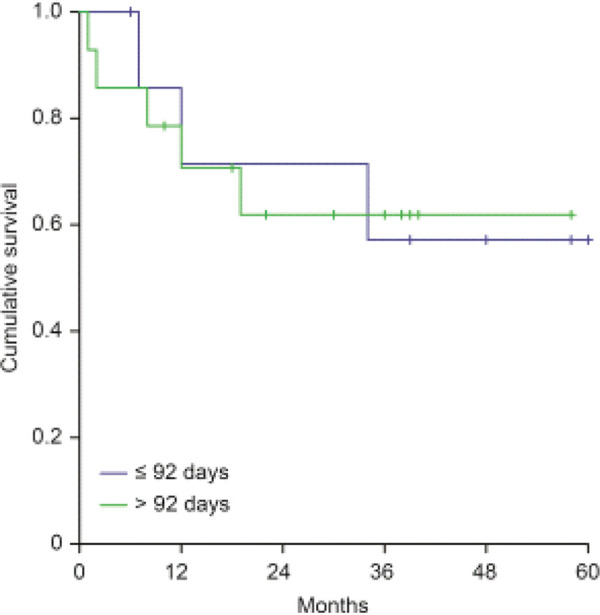
Kaplan-Meier curves of relapse-free cumulative survival according to time to start RT among patients with positive surgical margins.

**Figure 5 f05:**
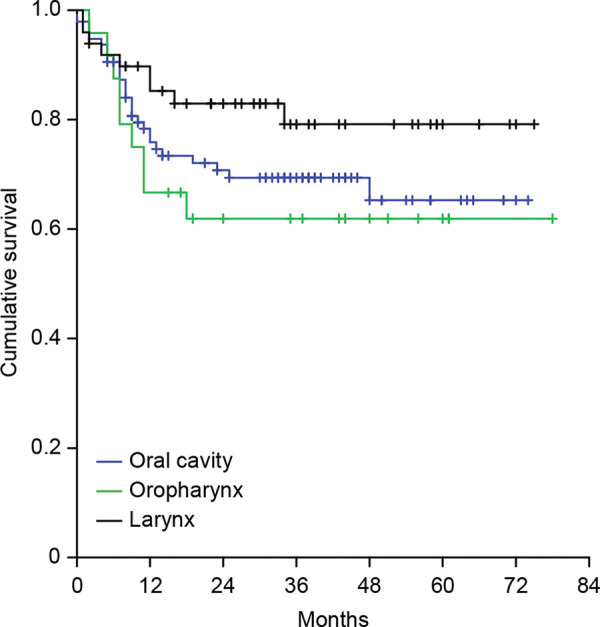
Kaplan-Meier curves of relapse-free survival according to the initial tumor site.

**Table 1:** Clinical, pathological and therapeutic features of patients.

Variable	N	%
Gender		
Male	132	78.6
Female	36	21.4
Primary Site		
Oral Cavity	95	56.5
Oropharynx	24	14.3
Larynx	49	29.2
Lymphovascular invasion	55	32.7
Perineural Invasion	119	70.8
Negative surgical margins	146	86.9
Positive lymph nodes	112	66.7
Extracapsular spread	55	32.7
Pathological Stage		
I-II	12	7.1
III-IV	156	92.9
Adjuvant Chemotherapy	80	47.6
Interval from surgery to RT		
<92 Days	48	26.6
>92 Days	120	71.4

**Table 2:** Univariate analysis of risk factors for locoregional recurrence.

Variable	Recurrences (number of events / patients)	Cumulative Progression Free Survival (%)	*p* *
Male gender	32/132	75.8	0.095
Female gender	14/36	61.1	
Primary site			
Larynx	9/49	81.6	0.096 (larynx vs. oropharynx)
Oropharynx	9/24	62.5	0.148 (larynx vs. oral cavity)
Oral cavity	28/95	70.5	0.096 (oropharynx vs. oral cavity)
Grade 1 or 2	36/143	74.8	0.130
Grade 3	9/24	62.5	
ALI	19/55	65.5	0.055
ALI absent	27/113	76.1	
PNI	42/119	64.7	<0.001
PNI absent	4/49	91.8	
Positive node	40/112	64.3	<0.001
Negative node	6/56	89.3	
ECE	21/55	61.8	0.014
ECE absent	25/113	77.9	
Stage II/III	8/39	79.5	0.187
Stage IV	38/129	70.5	
Timing surgery to RT consultation			
>63 days	24/70	65.7	0.118
<63 days	22/98	77.6	
Timing RT consultation to RT			
>22 days	37/153	75.8	0.002
<22 days	9/15	40.0	
Timing surgery to RT			
>92 days	35/120	70.8	0.397
<92 days	11/48	77.1	
Interruption during RT			
Yes	27/115	76.5	0.098
No	19/53	64.2	
Chemotherapy			
Yes	23/80	71.3	0.212
No	25/88	71.5	
Dose at surgical bed			<0.001 (<60Gy *vs*. 60Gy)
<60 Gy	7/168	12.5	<0.001 (<60Gy *vs*. 66Gy)
60Gy	14/168	78.4	0.003 (<60Gy *vs*. 70Gy)
66Gy	16/168	70.3	0.251 (60Gy *vs*.66Gy)
70Gy	9/168	55.9	0.008 (60Gy *vs*.70Gy)
			0.068 (66Gy *vs*. 70Gy)
BIM before RT			
<22.9 kg/m^2^	33/110	70.0	0.194
>22.9 kg/m^2^	13/57	77.2	
KPS before RT			
<70	14/29	51.7	0.001
>70	31/136	77.2	
KPS after RT			
<70	19/34	44.1	<0.001
>70	24/127	81.1	

* Log-rank test.

ALI = angiolymphatic invasion; BIM = body index mass; KPS = karnofsky performance status.

**Table 3:** Multivariate analysis of risk factors for locoregional recurrence.

Variable	HR	95% CI	*p*
KPS <70	2.058	1.060-3.992	0.033
Radiotherapy Length >130 Days	1.292	0.157-10.612	0.812
Dose at Surgical Bed <60 Gy	6.523	2.266-18.777	0.001
Radiotherapy Interruption	0.560	0.307-1.019	0.058
Regional Lymph Node Metastasis	3.339	1.350-8.255	0.009
Perineural Invasion	3.529	1.236-10.074	0.018
Extracapsular Extension	1.760	0.895-3.460	0.101
Male Gender	0.666	0.349-1.272	0.218
Angiolymphatic Invasion	1.105	0.573-2.131	0.766

HR = hazard ratio; CI = confidence interval.
